# Sleep and Prospective Memory: A Retrospective Study in Different Clinical Populations

**DOI:** 10.3390/ijerph17176113

**Published:** 2020-08-22

**Authors:** Lorenzo Tonetti, Miranda Occhionero, Michele Boreggiani, Andreas Conca, Paola Dondi, Maxime Elbaz, Marco Fabbri, Caroline Gauriau, Giancarlo Giupponi, Damien Leger, Monica Martoni, Chiara Rafanelli, Renzo Roncuzzi, Marina Zoppello, Vincenzo Natale

**Affiliations:** 1 Department of Psychology “Renzo Canestrari”, University of Bologna, 40127 Bologna, Italy; miranda.occhionero@unibo.it (M.O.); michele.boreggiani@gmail.com (M.B.); chiara.rafanelli@unibo.it (C.R.); vincenzo.natale@unibo.it (V.N.); 2 Division of Psychiatry, San Maurizio Hospital, 39100 Bolzano, Italy; andreas.conca@sabes.it (A.C.); giancarlo.giupponi@sabes.it (G.G.); 3 Division of Hospital Psychology, New Sant’Agostino-Estense Hospital, 41126 Baggiovara, Italy; dondi.paola@aou.mo.it; 4 Université Paris Descartes, APHP, Hôtel Dieu, Centre du Sommeil et de la Vigilance, Centre de référence hypersomnies rares et EA 7330 VIFASOM, 75004 Paris, France; maxime.elbaz@aphp.fr (M.E.); caroline.gauriau@aphp.fr (C.G.); damien.leger@aphp.fr (D.L.); 5 Department of Psychology, University of Campania “Luigi Vanvitelli”, 81100 Caserta, Italy; marco.fabbri@unicampania.it; 6 Department of Experimental, Diagnostic and Specialty Medicine, University of Bologna, 40127 Bologna, Italy; monica.martoni@unibo.it; 7 Cardiology Service, Villa Erbosa Hospital, 40129 Bologna, Italy; renzo.roncuzzi@libero.it; 8 Child Neuropsychiatry Unit, IRCCS Mondino Foundation, 27100 Pavia, Italy; marina.zoppello@gmail.com

**Keywords:** prospective memory, actigraphy, sleep, narcolepsy, primary insomnia, attention deficit hyperactivity disorder, obesity, essential hypertension, menopause

## Abstract

Prospective memory (PM) is essential in everyday life because it concerns the ability to remember to perform an intended action in the future. This ability could be influenced by poor sleep quality, the role of which, however, is still being debated. To examine the role of sleep quality in PM in depth, we decided to perform a retrospective naturalistic study examining different clinical populations with a primary sleep disorder or comorbid low sleep quality. If sleep is important for PM function, we could expect poor sleep to affect PM performance tasks both directly and indirectly. We examined a total of 3600 nights, recorded using actigraphy in participants belonging to the following groups: primary insomnia (731 nights); narcolepsy type 1 (1069 nights); attention deficit hyperactivity disorder (152 nights in children and 239 in adults); severe obesity (232 nights); essential hypertension (226 nights); menopause (143 nights); healthy controls (808 nights). In a naturalistic activity-based PM task, each participant originally wore an actigraph around the non-dominant wrist and was requested to push the event-marker button at two specific times of day: bedtime (activity 1) and get-up time (activity 2). Each clinical group showed significantly lower sleep quality in comparison to the control group. However, only narcolepsy type 1 patients presented a significantly impaired PM performance at get-up time, remembering to push the event-marker button around half the time compared not only to healthy controls but also to the other clinical groups. Overall, the present results seem to point to sleep quality having no effect on the efficiency of a naturalistic activity-based PM task. Moreover, the data indicated that narcolepsy type 1 patients may show a disease-specific cognitive deficit of PM.

## 1. Introduction

Prospective memory (PM) refers to the ability to remember to perform an action in the future [1]. An optimal functioning of PM is essential in everyday life [2], for example, in a patient who is required to take a drug every day at specified intervals. This example refers to the so-called habitual PM [3], a PM subdomain alongside vigilance (e.g., preventing a kettle from boiling over, as suggested by [3]) and PM proper (e.g., buying groceries on the way home, as proposed by [3]). Habitual PM can be assessed through a PM task that has to be executed repeatedly, while vigilance and PM proper can be assessed through tasks with the absence or the presence of a time delay (or intervening task) between PM task instructions and the beginning of the ongoing task, respectively [3].

It has been suggested [4,5] that two processes are involved in prospective remembering (remembering to perform a delayed intention in the future): (1) a top-down mechanism implicated in the strategic monitoring, i.e., maintaining the intention in memory while monitoring the environment in order to detect potential stimuli related to the intention; (2) a bottom-up mechanism involved in the spontaneous retrieval, which is spontaneously activated when an intention-related stimulus is detected.

The interaction of the two processes depends on the executive functions which regulate the distribution of cognitive load between ongoing activity and prospective intention. This sharing of resources is therefore considered to be primarily responsible for the interference effects underlying the prospective failure in everyday memory performance [6].

It has been hypothesized that these two processes are part of the Multiprocess Framework Model, originally proposed by McDaniel and Einstein in the early 2000s [4,5]. In 2013, Scullin and colleagues [7] proposed the Dynamic Multiprocess Framework; according to this framework, in the same prospective task, both strategic monitoring and spontaneous retrieval could be used, but with different timing and in separate contexts.

Three types of PM task have been described. (1) Time-based tasks [8], i.e., intentions to be performed at a specific time of day. Self-generated retrieval processes [9] are crucial in time-based tasks, which rely on top-down mechanisms due to the lack of external cues, with time monitoring representing a self-initiated mental process. (2) Event-based tasks [8], i.e., an external cue is necessary in order to spontaneously activate the intention to be performed [10]. Bottom-up mechanisms are involved in this specific PM task. (3) Activity-based tasks [11], i.e., an intention to be performed prior to or after an activity. Because activity-based PM tasks occur at the end of a task, they do not require the interruption of the ongoing cognitive load and thus are likely to be easier. A top-down and bottom-up mixed activation is required by this specific PM task. In particular, it is necessary that the external cue (bottom-up mechanism) is integrated with temporal monitoring (top-down process), requiring a mechanism of both internal and self-generated remembering.

The relationship between sleep quality and PM performance has attracted the interest of both sleep and cognitive scientists. The sleep literature has widely documented the relationship between sleep and executive function efficiency, demonstrating how sleep deprivation significantly compromises PM [12,13]. A recent systematic review and meta-analysis has been published on the effects of sleep on prospective memory [14]. In their work, Leong and colleagues [14] examined 20 studies overall, published between 2002 and 2019; some works investigated strategic monitoring while others explored spontaneous retrieval. The main finding of this systematic review and meta-analysis was, in general, a small-to-medium positive effect of sleep on PM. However, when strategic and spontaneous retrieval were considered separately, no effects of sleep were detected on strategic monitoring, but were, on the contrary, observed on the spontaneous retrieval process. Furthermore, when time- and event-based tasks were examined separately, a medium-sized effect of sleep was observed for time-based tasks, while a small effect was apparent for event-based tasks. Although not discussed by Leong and colleagues [14], a further potential source of inconsistency between the results of different studies in the association between sleep and PM could be the different focus on the three pillars of sleep, i.e., quality, quantity, and timing. Moreover, it is still not currently known which of the three sleep pillars could be more strongly associated with PM. Overall, this pattern of results highlights the fact that the role of low sleep quality in the impairment of PM performance is still a matter of debate, as also shown by a recent study [15] that failed to find a significant association between sleep and PM and which is not included in the above-mentioned systematic review and meta-analysis [14]. Confirming that the role of low sleep quality in the impairment of PM should still be clarified, a recent study of our research group [16] showed, in a large healthy lifespan sample, that sleep quality, quantity, and timing do not play a predictive role in PM performance contrary to aging that was per se associated with its worsening.

With the aim of objectively clarifying the role of sleep quality in habitual PM, we decided to perform a retrospective naturalistic study examining different clinical populations, with a primary sleep disorder or comorbid low sleep quality, alongside a sample of healthy controls. We chose to focus on habitual PM because its optimal functioning is of extreme relevance in everyday life, to the point of being considered a relevant component of medication adherence [17]. We used a naturalistic activity-based PM task: remembering to press the event-marker button on the top of the actigraph at bedtime (activity 1) and at get-up time (activity 2). This naturalistic activity-based PM task was firstly introduced by our research group [18,19], showing no differences in PM performance between primary insomnia patients and healthy controls. Later, the same task was adopted by the research group coordinated by Kinsella [20,21], showing the lack of a strong association between sleep and PM performance in a sample of community-dwelling older adults. This task is interesting for several reasons; it is simple but of great importance for the participants and also allows for an objective evaluation of the executive component of prospective memory. If sleep per se has an effect on PM, we should be able to observe this effect regardless of the different clinical features of the populations examined, in other words, we should expect a worsening in PM performance proportional to the sleep impairment. More specifically, if low sleep quality, a characteristic of each clinical population, plays a major role in the deterioration of PM, we could expect: (1) an impaired PM performance at get-up time in each clinical group in comparison to healthy controls; (2) a highly impaired PM performance at get-up time compared to bedtime within each clinical population; (3) a correlation between poor sleep and impaired PM performance at get-up time within each clinical sample.

## 2. Materials and Methods

### 2.1. Participants

Three hundred and sixty participants (206 females and 154 males), actigraphically monitored for a total of 3600 nights, were examined in this retrospective study.

They belonged to the following groups: (1) childhood attention deficit hyperactivity disorder (C-ADHD) [22]: 22 patients, four females and 18 males, mean age ± SD = 8.77 ± 1.77, 152 actigraphy-recorded nights; (2) healthy controls (HC) [23,24,25]: 95 participants, 57 females and 38 males, mean age ± SD = 27.93 ± 14.35, 808 actigraphy-recorded nights; (3) adult ADHD (A-ADHD) [26]: 35 patients, 14 females and 21 males, mean age ± SD = 37.29 ± 12.23, 239 actigraphy-recorded nights; (4) narcolepsy type 1 (NT1) [27]: 40 patients, 28 females and 12 males, mean age ± SD = 39.7 ± 10.96, 1069 actigraphy-recorded nights; (5) severe obesity (SO) [28]: 34 patients, 18 females and 16 males, mean age ± SD = 45.79 ± 9.43, 232 actigraphy-recorded nights; (6) primary insomnia (PI) [23]: 82 patients, 49 females and 33 males, mean age ± SD = 49.69 ± 21.44, 731 actigraphy-recorded nights; (7) menopause (MP) (unpublished data): 21 women, mean age ± SD = 54.55 ± 5.19, 143 actigraphy-recorded nights; (8) essential hypertension (HP) [28]: 31 patients, 15 females and 16 males, mean age ± SD = 63 ± 8.54, 226 actigraphy-recorded nights.

In order to clarify the role of sleep quality in PM, we chose to retrospectively examine these clinical populations because they presented a primary sleep disorder (i.e., NT1 and PI) or comorbid low sleep quality (i.e., the remaining clinical groups).

### 2.2. Actigraphy

All participants used the same model of actigraph, the Actiwatch AW-64 (Cambridge Neurotechnology Ltd., Cambridge, UK). This device is equipped with an accelerometer which is sensitive to movement. Motor activity data are then transformed into sleep/wake information using a validated algorithm [29]. This device has been successfully validated against polysomnography (e.g., [30]), the gold standard for sleep assessment.

The hardware of this device is composed of a piezoelectric accelerometer presenting a sensitivity of ≥0.05 g. Filters were set to 3–11 Hz, with sampling frequency equal to 32 Hz. Actiwatch Activity and Sleep Analysis software (version 5.32, Cambridge Neurotechnology Ltd., Cambridge, UK) was used in order to initialize actigraphs to collect data in 1-min epochs.

Actigraphy is particularly useful in the case of long-term home monitoring, when information on sleep stage is not of primary importance [31].

### 2.3. Procedure of the Original Studies

Each participant was originally requested to wear the actigraph around the non-dominant wrist for at least seven consecutive days. Due to logistic reasons, some participants wore the actigraph for a period lower or higher than the standard of 7 days, resulting in a mean length of actigraphy recording equal to 10 (standard deviation = 6.91) consecutive days. For each participant, the whole recording period, regardless of its composition in terms of weekdays and/or weekend days, was examined. They were required to push the event-marker button on the actigraph in order to signal: (1) the time of day they went to bed in order to attempt to sleep, bedtime (BT); (2) the time of day they got out of bed at the end of a night’s sleep, get-up time (GUT). Within 30 min of morning awakening, participants had to fill in a daily sleep log, which also included questions on BT and GUT.

Using the low wake sensitivity threshold [23,30] implemented within the Actiwatch Activity and Sleep Analysis software (version 5.32, Cambridge Neurotechnology Ltd., Cambridge, UK), an expert scorer analyzed the actigraphic recordings using the information on BT and GUT provided by the participants’ pressing of the event-marker button of the actigraph. If participants failed to press the event-marker button, the scorer referred to their replies to the questions on BT and GUT reported in the sleep log.

Adult participants gave written informed consent prior to inclusion in the original studies; if underage, written informed consent was provided by parents. Original studies were approved by the Ethics Committee in charge.

### 2.4. Actigraphic Measures

Through actigraphic recording, we were able to objectively monitor sleep timing, quantity, and quality. A description of the actigraphic parameters used to assess these three different aspects of sleep is reported below.

Sleep timing was assessed by the midpoint of sleep (MS), i.e., the clock time that splits the interval between BT and GUT, i.e., the time in bed (TIB), in half. Sleep quantity was investigated by the total sleep time (TST), i.e., the sum in minutes of sleep epochs between sleep onset (SO) and GUT. Sleep quality was examined through the wake after sleep onset (WASO; the sum in minutes of wake epochs between SO and GUT), sleep onset latency (SOL; the interval in minutes between BT and SO), sleep efficiency (SE; the ratio between TST and TIB multiplied by 100), mean activity score (MAS; mean value of activity counts per epoch over the assumed sleep period), and fragmentation index (FI, the percentage of immobility phases of 1 min as a proportion of the total number of immobility phases).

### 2.5. Activity-Based Prospective Memory Task

We considered the request received by all participants to remember to push the event-marker button at bedtime and get-up time as the activity-based prospective memory task. In particular, the pushing of the event-marker button at bedtime and get-up time corresponded to activity 1 and 2, respectively.

### 2.6. Procedure of the Current Study

With reference to the activity-based prospective memory performance, in the current study, each actigraphic recording was visually inspected night by night in order to verify whether participants remembered to press the event-marker button at BT—activity 1—and GUT—activity 2. In order to validate the PM performance for activity 2, we implemented a limit of 15 min between the GUT and the time activity 2 was performed.

### 2.7. Statistical Analyses

First, with the aim of exploring differences between groups in sleep timing, quality, and quantity, we carried out some pair comparisons between HC and each clinical sample, through a set of independent sample *t*-tests with group as the independent variable (two levels: HC and clinical group) and actigraphic sleep parameter as the dependent variable.

In order to explore the potential effects of a night’s sleep on prospective memory performance, firstly, we carried out an ANOVA with group as the independent variable (eight levels, each one corresponding to the group examined) and the percentage of successful event-marker button pressing at GUT subjected to arcsine transformation as the dependent variable. In the case of a significant effect, Tukey’s honestly significant difference (HSD) post hoc test for unequal samples was performed. Arcsine transformation was applied to correct the non-normal distribution of percentage values. Secondly, we compared, separately for each group, the prospective memory performance at BT (activity 1) with that at GUT (activity 2) through dependent sample *t*-tests. Thirdly, we performed a set of point-biserial correlation analyses, separately for each group, between each actigraphic sleep parameter and PM performance at GUT (activity 2).

Since multiple comparisons were performed, the Bonferroni correction was applied, considering *p*-values less than 0.001 as significant.

## 3. Results

### 3.1. Actigraphic Sleep Parameters

Table 1 shows the means and standard deviations of actigraphic sleep parameters in each group, while Table 2 illustrates the statistics for each pair comparison between HC and clinical samples. As shown in Table 2, all clinical groups significantly differed from HC by a minimum of four to a maximum of six parameters.

### 3.2. Sleep Effect on Prospective Memory Performance

**Figure 1:**
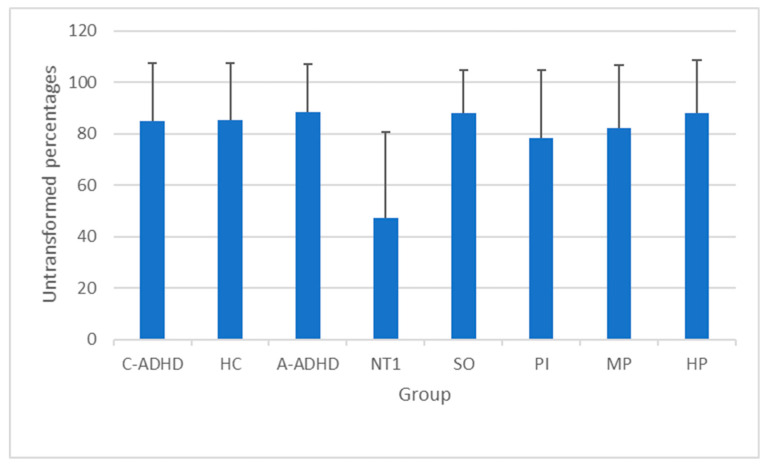
Means and standard deviations of prospective memory performance at get-up time in each group. In order to present the results more clearly, we have chosen to report the untransformed percentages in the figure, even though the statistical analyses were performed on the percentages subjected to arcsine transformation. C-ADHD = childhood attention deficit hyperactivity disorder; HC = healthy controls; A-ADHD = adult attention deficit hyperactivity disorder; NT1 = narcolepsy type 1; SO = severe obesity; PI = primary insomnia; MP = menopause; HP = essential hypertension.

Prospective memory performance at get-up time was significantly different between groups (F_7352_ = 12.65; *p* = 0.000) (Figure 1). When post hoc comparisons were performed, NT1 patients significantly differed from all the remaining groups (*p* = 0.000 for each comparison). No other post hoc comparisons reached significance.

No significant differences were observed when we performed a within-group comparison between prospective memory performance at bedtime (activity 1) and get-up time (activity 2) (see Table 3).

When we performed a set of within-group point-biserial correlation analyses between each actigraphic sleep parameter and PM performance at get-up time (activity 2), we observed the following significant correlations in the NT1 sample (see Table 4): negative correlations between WASO, MAS, FI, and MS and PM performance; a positive correlation between SE and PM performance.

## 4. Discussion

The main aim of the present study was to explore the potential role of sleep quality and amount in PM, which is currently still a matter of debate [14,15,16]. To this end, we carried out a retrospective naturalistic study in which we examined different clinical populations with a primary sleep disorder or comorbid low sleep quality. In more detail, we compared the PM performance of these groups in a naturalistic activity-based PM task [18,19,20,21]. If sleep played a primary role in the modulation of PM, we expected to find an impaired PM performance at get-up time in each clinical group compared to healthy controls. Furthermore, such a sleep effect (if any) should translate, within each clinical population, into a worse PM performance at get-up time compared to bedtime, as well as into significant correlations between poor sleep and PM performance at get-up time.

With regard to actigraphy-measured sleep, as shown in Table 2, we observed three homogeneous patterns of results between different clinical populations, i.e., lower sleep efficiency, as well as higher mean activity score and fragmentation index (all markers of sleep quality), compared to healthy controls.

These data seem to point out that each clinical population significantly differed from healthy controls in terms of their impaired sleep quality. This pattern of results is in line with the currently available knowledge on sleep quality in these clinical populations [32,33,34,35,36,37,38], confirming the representativeness of our sample. 

In spite of these significant differences between clinical groups and healthy controls in actigraphy-measured sleep, when we examined the prospective memory performance at get-up time (Figure 1), we observed a significantly impaired PM performance only in narcolepsy type 1 patients compared to each of the remaining groups. Moreover, as reported in Table 3, we tried to carry out a more thorough investigation into a potential sleep effect on PM by comparing performance in the activity-based PM task at bedtime with that at get-up time, separately for each group; however, we did not observe any significant differences. Finally, as shown in Table 4, only in narcolepsy type 1 patients did we observe significant associations between impaired sleep quality and delayed sleep timing on one hand, and a weakened PM performance at get-up time on the other. These three patterns of results are completely in disagreement with the expectations we put forward, based on the supposition that low sleep quality, that characterizes each clinical population, could play a major role in the deterioration of PM.

Therefore, bearing in mind the differences between groups in actigraphy-measured sleep (Table 2), the pattern of results reported in Figure 1 and Table 3 and Table 4 seems to smooth the potential modulating role of sleep quality on PM, at least in this specific type of PM task carried out in a naturalistic setting. A possible explanation for the impaired PM performance in narcolepsy type 1 patients at get-up time is more likely related to a sort of disease-specific cognitive deficit of PM in these patients, regardless of sleep quality. An additional explanation could be that in narcolepsy the functional importance of the sleep architecture is lacking, and this aspect may have important effects on memory processes [39]. To the best of our knowledge, only Ohayon and colleagues [40] have examined the self-referred PM deficits in narcolepsy patients, asking them to fill in the Cognitive Difficulties Scale [41] which also included a dimension on PM cognitive difficulty. The authors found that, when controlling for some potential confounding factors (age, ongoing psychotropic medication treatment, sleep apnea, physical disease, and sleepiness), narcolepsy remained significantly related to attention–concentration and PM deficits. This pattern of results would seem to suggest that PM deficits in narcolepsy patients cannot be explained by factors other than narcolepsy itself. A review by Cipolli and colleagues [42] highlighted a sleep-dependent consolidation impairment in narcolepsy–cataplexy only for visual and procedural skills, the latter being pertinent to PM. In fact, prospective memory involves both the episodic memory system (content of the intention) and executive processes (acting upon the intention). Mazzetti and colleagues [43] observed difficulties in the consolidation of both visual discrimination and motor skills (procedural memory system) in narcoleptic patients. Another specificity of narcolepsy is represented by the abnormalities in rapid eye movement sleep [44]; the decrease in rapid eye movement sleep length with aging has been suggested to mediate the age-related PM decline in healthy adults [45]. However, a recent review highlights how data on the specific components of memory in relation to sleep are still controversial, and further and more precise research is needed in this regard [46]. As Stickgold [47] points out, “At the very least, one has to address the question of how five stages of sleep interact with at least six types of memories and six stages of post-encoding memory processing, for a combined total of 144 distinct sub-questions” (page 305).

We acknowledge some limitations of the present study. First, we examined just one type of PM task, i.e., the activity-based PM task, so our conclusions cannot be generalized to the other two types of PM task (time- and event-based). Second, since we retrospectively examined clinical groups, their sizes could not be defined a priori, thus resulting in samples that were unbalanced in size. Third, not all clinical groups were drug free, and therefore potential side effects on sleep and PM may have been present. Fourth, polysomnographic recording was lacking; this technique would have allowed us to analyze the sleep architecture and examine in more depth the potential association between slow wave [48] and rapid eye movement [45] sleep on one hand and prospective memory on the other. Fifth, due to the naturalistic setting of our study, we were not able to check the use of external aids to remember to perform the activity-based PM task.

This study also presents some strengths, such as the impressively large number of actigraphically recorded nights (3600), the naturalistic setting in which participants performed the activity-based PM task, and the objective assessment of sleep quality, quantity, and timing through actigraphy.

Future studies should try to examine the PM performance in time, as well as event-based PM tasks in a naturalistic setting, by prospectively enrolling a balanced number of drug-free patients belonging to different clinical populations with a primary sleep disorder or comorbid low sleep quality.

## 5. Conclusions

The present study contributes to the still open research question about the potential role of sleep quality in the modulation of PM. The main finding is that, despite impaired sleep quality in each clinical group compared to healthy controls, only narcolepsy type 1 patients presented a weakened PM performance at get-up time in comparison to the remaining groups. This pattern of results allows us to conclude that, at least for an activity-based PM task carried out in a naturalistic setting, the role of sleep quality does not seem to be crucial. On the contrary, the results observed seem to point to a disease-specific cognitive deficit of PM in narcolepsy type 1.

## Figures and Tables

**Table 1:** Means and standard deviations of actigraphy-measured sleep parameters of different groups of patients and healthy controls, listed in increasing order of average age.

Actigraphy-Measured Sleep Parameter	C-ADHD	HC	A-ADHD	NT1	SO	PI	MP	HP
MS	03:12 ± 0:33	04:17 ± 01:23	03:58 ± 1:19	04:37 ± 1:49	03:55 ± 0:58	03:58 ± 1:06	03:07 ± 0:50	03:31 ± 1:18
TST	468.33 ± 36.03	405.52 ± 44.44	420.02 ± 61.31	411.48 ± 61.95	362.04 ± 69.04	435.75 ± 55.67	401.74 ± 54.97	390.84 ± 44.03
WASO	82.22 ± 27.55	27.98 ± 9.96	44.03 ± 18.95	45.82 ± 28.48	39.45 ± 25	47.71 ± 19.77	39.14 ± 14.27	37.38 ± 19.99
SOL	18.04 ± 9.26	6.48 ± 3.90	15.17 ± 12.42	10.80 ± 8.82	25.20 ± 27.88	13.88 ± 11	7.71 ± 7.07	14.53 ± 11.49
SE	81.40 ± 5.05	91.45 ± 2.64	86.96 ± 4.31	86.89 ± 7.47	83.43 ± 10.75	86.67 ± 5.11	88.73 ± 3.97	87.23 ± 5.12
MAS	22.50 ± 16.27	11.92 ± 3.50	20.42 ± 9.74	20.06 ± 11.86	21.82 ± 15.82	21 ± 10.17	18.97 ± 7.27	19.62 ± 11.93
FI	34.03 ± 7.32	21.91 ± 4.94	33 ± 8.07	42.17 ± 15.56	41.85 ± 26.05	31.33 ± 8.65	30.03 ± 8.75	33.51 ± 10.40

*Legend.* MS = midpoint of sleep (h:min); TST = total sleep time (min); WASO = wake after sleep onset (min); SOL = sleep onset latency (min); SE = sleep efficiency (%); MAS = mean activity counts (activity counts); FI = fragmentation index (sum of percentages). Groups included: C-ADHD = childhood attention deficit hyperactivity disorder; HC = healthy controls; A-ADHD = adult attention deficit hyperactivity disorder; NT1 = narcolepsy type 1; SO = severe obesity; PI = primary insomnia; MP = menopause; HP = essential hypertension.

**Table 2:** Pair comparisons between healthy controls (HC) and patients from several clinical samples.

Actigraphy-Measured Sleep Parameter	C-ADHD vs. HC (*t_115_*)	A-ADHD vs. HC (*t_128_*)	NT1 vs. HC (*t_133_*)	SO vs. HC (*t_127_*)	PI vs. HC (*t_175_*)	MP vs. HC (*t_114_*)	HP vs. HC (*t_124_*)
MS	3.55; *p* = 0.001	1.18; *p* = 0.239	−1.18; *p* = 0.241	1.43; *p* = 0.154	1.63; *p* = 0.105	**3.71; *p* = 0.000**	2.69; *p* = 0.008
TST	**−6.17; *p* = 0.000**	−1.48; *p* = 0.141	−0.63; *p* = 0.530	**4.19; *p* = 0.000**	**−4.01; *p* = 0.000**	0.34; *p* = 0.736	1.60; *p* = 0.112
WASO	**−15.47; *p* = 0.000**	**−6.26; *p* = 0.000**	**−5.40; *p* = 0.000**	**−3.74; *p* = 0.000**	**−8.55; *p* = 0.000**	**−4.27; *p* = 0.000**	−3.47; *p* = 0.001
SOL	**−9.22; *p* = 0.000**	**−6.09; *p* = 0.000**	**−3.96; *p* = 0.000**	**−6.42; *p* = 0.000**	**−6.13; *p* = 0.000**	−1.11; *p* = 0.271	**−5.91; *p* = 0.000**
SE	**13.19; *p* = 0.000**	**7.16; *p* = 0.000**	**5.24; *p* = 0.000**	**6.76; *p* = 0.000**	**7.96; *p* = 0.000**	**3.87; *p* = 0.000**	**5.98; *p* = 0.000**
MAS	**−5.85; *p* = 0.000**	**−7.35; *p* = 0.000**	**−6.11; *p* = 0.000**	**−5.76; *p* = 0.000**	**−8.17; *p* = 0.000**	**−6.64; *p* = 0.000**	**−5.63; *p* = 0.000**
FI	**−9.40; *p* = 0.000**	**−9.45; *p* = 0.000**	**−11.45; *p* = 0.000**	**−7.16; *p* = 0.000**	**−9.05; *p* = 0.000**	**−5.82; *p* = 0.000**	**−8.39; *p* = 0.000**

*Legend.* MS = midpoint of sleep; TST = total sleep time; WASO = wake after sleep onset; SOL = sleep onset latency; SE = sleep efficiency; MAS = mean activity counts; FI = fragmentation index. Groups included: C-ADHD = childhood attention deficit hyperactivity disorder; HC = healthy controls; A-ADHD = adult attention deficit hyperactivity disorder; NT1 = narcolepsy type 1; SO = severe obesity; PI = primary insomnia; MP = menopause; HP = essential hypertension. The *t*- and *p*-values, referring to each pair, are reported, with significant effects highlighted in bold.

**Table 3:** Means and standard deviations of prospective memory performance at bedtime (activity 1) and get-up time (activity 2). Although statistical analyses were performed on arcsine-transformed percentages, we chose to report the untransformed percentage values in the table. Statistics are also reported.

Group	BT	GUT	Statistics
C-ADHD	79.61 ± 25.16	85.06 ± 22.25	*t_21_* = 0.90; *p* = 0.381
HC	83.92 ± 24.16	85.31 ± 22.28	*t_94_* = 0.52; *p* = 0.606
A-ADHD	83.82 ± 20.20	88.45 ± 18.63	*t_34_* = 1.27; *p* = 0.213
NT1	50.90 ± 30.83	47.14 ± 33.34	*t_39_* = 0.91; *p* = 0.367
SO	82.63 ± 22.87	87.89 ± 16.97	*t_33_* = 1.21; *p* = 0.233
PI	81.19 ± 19.21	78.50 ± 26.11	*t_81_*= 0.78; *p* = 0.440
MP	75.62 ± 32.58	82.09 ± 24.42	*t_20_* = −0.80; *p* = 0.433
HP	86.94 ± 20	88.15 ± 20.29	*t_30_* = −0.53; *p* = 0.603

*Legend.* BT = bedtime; GUT = get-up time; C-ADHD = childhood attention deficit hyperactivity disorder; HC = healthy controls; A-ADHD = adult attention deficit hyperactivity disorder; NT1 = narcolepsy type 1; SO = severe obesity; PI = primary insomnia; MP = menopause; HP = essential hypertension.

**Table 4:** Point-biserial correlations, within each group, between each actigraphic sleep parameter and PM performance at get-up time (activity 2).

Actigraphy-Measured Sleep Parameter	C-ADHD	HC	A-ADHD	NT1	SO	PI	MP	HP
MS	−0.100; *p* = 0.220	0.026; *p* = 0.462	−0.201; *p* = 0.002	**−0.183; *p* = 0.000**	−0.053; *p* = 0.420	−0.099; *p* = 0.007	0.005; *p* = 0.956	0.005; *p* = 0.935
TST	0.055; *p* = 0.501	−0.001; *p* = 0.985	−0.099; *p* = 0.126	0.059; *p* = 0.055	0.104; *p* = 0.113	−0.028; *p* = 0.452	0.045; *p* = 0.592	−0.092; *p* = 0.169
WASO	−0.045; *p* = 0.580	−0.021; *p* = 0.559	−0.023; *p* = 0.718	**−0.329; *p* = 0.000**	0.061; *p* = 0.357	−0.036; *p* = 0.325	−0.127; *p* = 0.130	0.033; *p* = 0.622
SOL	0.003; *p* = 0.971	−0.034; *p* = 0.330	−0.079; *p* = 0.226	−0.047; *p* = 0.124	−0.183; *p* = 0.005	−0.051; *p* = 0.165	−0.226; *p* = 0.007	−0.083; *p* = 0.214
SE	0.002; *p* = 0.977	0.034; *p* = 0.331	0.014; *p* = 0.827	**0.265; *p* = 0.000**	0.113; *p* = 0.087	0.037; *p* = 0.320	0.144; *p* = 0.087	0.030; *p* = 0.649
MAS	0.003; *p* = 0.973	−0.085; *p* = 0.016	0.019; *p* = 0.768	**−0.327; *p =* 0.000**	−0.009; *p* = 0.893	0.005; *p* = 0.888	−0.122; *p* = 0.148	0.053; *p* = 0.430
FI	−0.031; *p* = 0.708	0.027; *p* = 0.449	−0.034; *p* = 0.599	**−0.273; *p =* 0.000**	−0.046; *p* = 0.484	0.028; *p* = 0.456	−0.105; *p* = 0.213	−0.006; *p* = 0.933

*Legend*. MS = midpoint of sleep; TST = total sleep time; WASO = wake after sleep onset; SOL = sleep onset latency; SE = sleep efficiency; MAS = mean activity counts; FI = fragmentation index. Groups included: C-ADHD = childhood attention deficit hyperactivity disorder; HC = healthy controls; A-ADHD = adult attention deficit hyperactivity disorder; NT1 = narcolepsy type 1; SO = severe obesity; PI = primary insomnia; MP = menopause; HP = essential hypertension. R and *p*-values are shown, with significant correlations highlighted in bold.
